# Small Molecule Fisetin Modulates Alpha–Synuclein Aggregation

**DOI:** 10.3390/molecules26113353

**Published:** 2021-06-02

**Authors:** Rita Rosado-Ramos, Joana Godinho-Pereira, Daniela Marques, Inês Figueira, Tiago Fleming Outeiro, Regina Menezes, Cláudia Nunes dos Santos

**Affiliations:** 1 iBET, Instituto de Biologia Experimental e Tecnológica, Av. da República, Apartado 12, 2781-901 Oeiras, Portugal; rramos@ibet.pt (R.R.-R.); joanagpereira@ff.ulisboa.pt (J.G.-P.); ifigueira@farm-id.pt (I.F.); regina.menezes@nms.unl.pt (R.M.); 2 Instituto de Tecnologia Química e Biológica António Xavier, Universidade Nova de Lisboa, Av. da República, 2780-157 Oeiras, Portugal; 3 CEDOC, NOVA Medical School, Faculdade de Ciências Médicas, Universidade NOVA de Lisboa, Campo dos Mártires da Pátria, 130, 1169-056 Lisboa, Portugal; daniela.marques@nms.unl.pt; 4 Department of Experimental Neurodegeneration, Center for Biostructural Imaging of Neurodegeneration, University Medical Center Göttingen, 37073 Göttingen, Germany; touteir@gwdg.de; 5 Max Planck Institute for Experimental Medicine, 37075 Göttingen, Germany; 6 Translational and Clinical Research Institute, Faculty of Medical Sciences, Newcastle University, Framlington Place, Newcastle Upon Tyne NE2 4HH, UK; 7 Scientific Employee with a Honorary Contract at Deutsches Zentrum für Neurodegenerative Erkrankungen (DZNE), 37075 Göttingen, Germany; 8 CBIOS—Universidade Lusófona’s Research Center for Biosciences & Health Technologies, Campo Grande 376, 1749-024 Lisboa, Portugal

**Keywords:** α-synuclein, dopamine transporter, flavonoid, Parkinson’s Disease

## Abstract

Phenolic compounds are thought to be important to prevent neurodegenerative diseases (ND). Parkinson’s Disease (PD) is a neurodegenerative disorder known for its typical motor features, the deposition of α-synuclein (αsyn)-positive inclusions in the brain, and for concomitant cellular pathologies that include oxidative stress and neuroinflammation. Neuroprotective activity of fisetin, a dietary flavonoid, was evaluated against main hallmarks of PD in relevant cellular models. At physiologically relevant concentrations, fisetin protected SH-SY5Y cells against oxidative stress overtaken by *tert*-butyl hydroperoxide (*t*-BHP) and against methyl-4-phenylpyridinuim (MPP^+^)-induced toxicity in dopaminergic neurons, the differentiated Lund human Mesencephalic (LUHMES) cells. In this cellular model, fisetin promotes the increase of the levels of dopamine transporter. Remarkably, fisetin reduced the percentage of cells containing αsyn inclusions as well as their size and subcellular localization in a yeast model of αsyn aggregation. Overall, our data show that fisetin exerts modulatory activities toward common cellular pathologies present in PD; remarkably, it modulates αsyn aggregation, supporting the idea that diets rich in this compound may prove beneficial.

## 1. Introduction

Parkinson’s disease (PD) is the most common neurodegenerative movement disorder affecting ~2% of the people above 65 years old [1]. It is characterized by the loss of dopaminergic neurons at the *substantia nigra pars compacta* and by the deposition of proteinaceous inclusions known as Lewy Bodies (LBs) and Lewy neurites in the surviving neurons. LBs are mainly formed by misfolded and aggregated α-synuclein (αsyn). Aggregation of αsyn is described to affect dopamine metabolism, to increase oxidative stress due to mitochondrial dysfunction, to disrupt synaptic function, and to impair vesicular trafficking [2]. In addition, the presence of activated immune cells, such as microglia and peripheral lymphocytes, in the *substantia nigra* of PD patients underpins the role of inflammation on disease progression [3].

Major research efforts have been made to understand the molecular basis of PD, with the goal of developing therapies to delay disease progression. However, the complexity of the disease has prevented the advance of long-lasting effective treatments [4]. Therefore, PD still represents a huge social and economic burden. Epidemiological and clinical studies have shown that dietary (poly)phenols can reduce the incidence of disorders such as PD [5] by the modulation of several molecular pathways [6,7]. Berry fruits comprise one of the richest sources of dietary flavonoids, and strawberry and blueberry intake has already been associated with lower risk of PD development, particularly in men [6]. Thus, diet modification might provide a solution to be used either as preventive or possibly as therapeutic co-adjuvants for PD. 

Fisetin is a flavonol found in strawberries, grapes, apples, persimmon, onion, and cucumber at concentrations in the range of 2–160 μg/g of fresh food [8]. Flavonols are extensively reported to exert health-promoting activities against various pathologies, including inflammation, diabetes, cancer, heart disease, and viral infections. Quercetin, kaempferol, and myricetin are the most studied flavonols due to their abundance in diet [9]. Moreover, fisetin is also described to play a protective role against several disease hallmarks, including neurodegeneration [10,11] and inflammation [12]. Detection of free and bioavailable circulating fisetin has been described following both intravenous and oral administration. Glucuronidated, sulfated, and methylated metabolites of fisetin are also detected in circulation [13,14]. Moreover, fisetin is also found in the brain of mice following oral and intraperitoneal administration [15], reinforcing the potential of this dietary (poly)phenol for brain health.

Fisetin is described to target critical pathological processes of brain disease such as oxidative stress and neuroinflammation [16]. In addition, fisetin is shown to induce neurite outgrowth through extracellular signal-regulated kinase 1/2 (ERK1/2) [10], to reduce tumor necrosis factor-α (TNF-α) levels and nitric oxide production, to downregulate nuclear factor Kappa B (NF-κB) and mitogen activator protein kinase (MAPK) pathways in microglia [12], and also to enhance learning and memory in WT mice [17], as reviewed by Maher, P. [11]. In PD, fisetin was shown to decrease cell death, to reduce αsyn levels, and to inhibit inflammatory and apoptotic markers in undifferentiated PC12 cells exposed to 1-methyl-4-phenyl-1,2,3,6-tetrahydropyridine (MPTP)/MPP^+^ [18]. Additionally, fisetin interacts with misfolded and aggregated huntingtin, a protein associated with Huntington’s disease [10]. However, there is still a knowledge gap on the molecular mechanisms underlying fisetin activity in fully differentiated human dopaminergic neurons and toward human αsyn aggregation. 

Different models have been used to study the underlying mechanisms of both disease pathology and small molecules activity. Yeast cells overexpressing αsyn have been extensively used for this purpose due to their many advantages. It is described that αsyn overexpression leads to αsyn aggregation and toxicity and to the recapitulation of the main cellular hallmarks of PD such as vesicular trafficking impairment and mitochondrial damages [19].

Our study aimed at understanding the mechanisms by which fisetin exerts neuroprotective action in PD. To achieve this purpose, physiologically relevant concentrations of fisetin (comparable to circulating levels) [14,20] were tested in a human cell model of cellular pathologies found in PD. In addition, we took advantage of a well-characterized yeast model of synucleinopathy [19,21,22] to study the effect of fisetin on αsyn aggregation. Our data show that fisetin confers protection against tert-butyl hydroperoxide (*t*-BHP)-mediated oxidative stress in SH-SY5Y cells and against MPP^+^ toxicity in differentiated LUHMES cells through the modulation of dopamine transporter (DAT) expression. Moreover, we show for the first time that fisetin reduces the percentage of yeast cells displaying αsyn inclusions as well as their size. Overall, our study reinforces the protective action of fisetin toward common pathological process associated with neurodegeneration as well as reveals the ability of fisetin to interfere with the accumulation of αsyn inclusions.

## 2. Results

### 2.1. Physiologically Relevant Concentrations of Fisetin Protect Neuronal Cells against Oxidative Insults

The neuroprotective action of fisetin was evaluated in human neuronal cell models submitted to oxidative insults. SH-SY5Y neuroblastoma cells exposed to *t*-BHP and differentiated LUHMES cells insulted with MPP^+^ were used as models. *t*-BHP was used as an oxidant stimulus whereas MPP^+^ is known to cause oxidative bursts by interfering with oxidative phosphorylation in mitochondria through the inhibition of complex I, leading to the depletion of adenosine triphosphate (ATP). In both cellular models, cell viability is compromised by the respective insults, and fisetin neuroprotective effects was evaluated.

**Figure 1:**
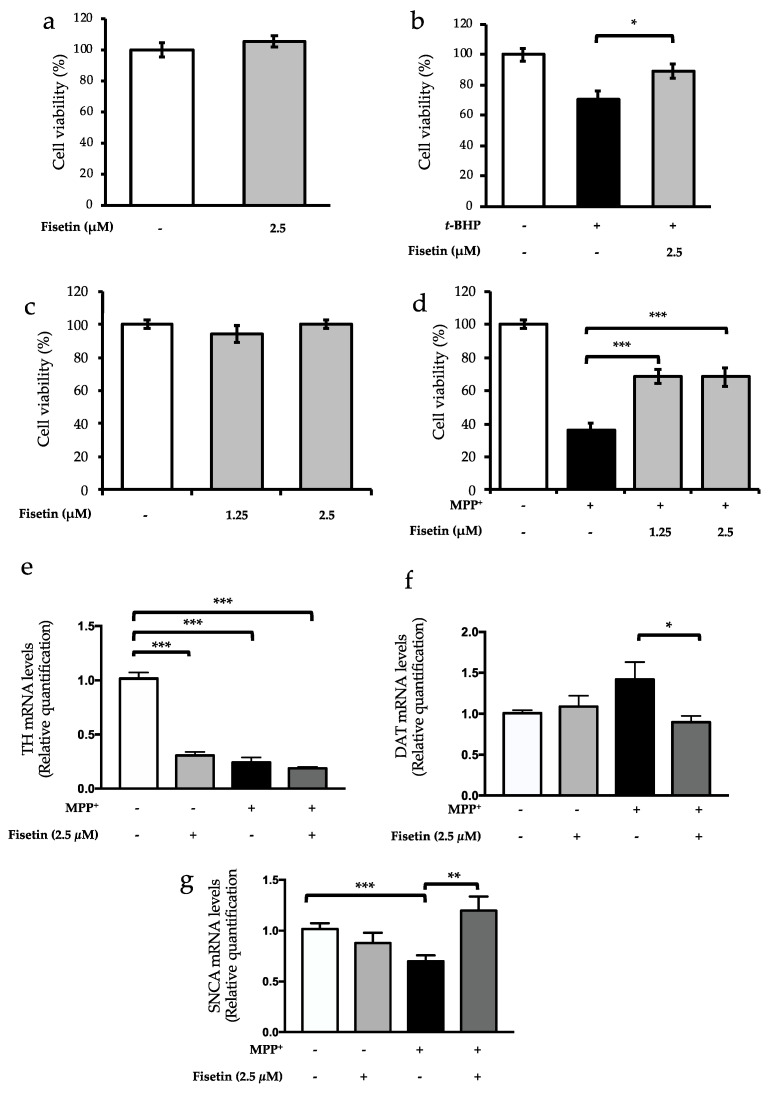
Fisetin rescues neuronal cells from oxidative insults. (**a**) SH-SY5Y cells were treated or not with 2.5 µM of fisetin for 6 h, and cell viability was assessed using CellTiter Blue^®^ Cell Viability Assay kit. (**b**) SH-SY5Y cells were pretreated or not with 2.5 µM of fisetin for 6 h and then challenged with 35 µM of *t*-BHP for 18 h. (**c**) LUHMES cells were treated or not with fisetin (1.25 or 2.5 µM) for 24 h, and cell viability was assessed using CellTiter Blue^®^ Cell Viability Assay kit. (**d**) LUHMES cells were pretreated or not with fisetin (1.25 or 2.5 µM) for 24 h and then challenged with 5 µM of MPP^+^ for 24 h. Cell viability was assessed using CellTiter Blue^®^ Cell Viability Assay kit. (**e**) Tyrosine hydroxylase (TH), (**f**) dopamine transporter (DAT), and (**g**) α-synuclein (SNCA) mRNA levels in LUHMES were assessed by qRT-PCR using HPRT1, GAPDH, and B2M as reference genes. The values represent the mean ± SEM of at least three biological replicates. Statistic differences are denoted by * *p* < 0.05, ** *p* < 0.01, and *** *p* < 0.001 vs. indicated condition.

The pretreatment of SH-SY5Y cells with 2.5 µM of fisetin, a concentration within the physiological circulating range of (poly)phenols [14,20,23], did not present any toxic effect (Figure 1a). Chronic oxidative insult was mimicked by exposing cells to 35 µM of *t*-BHP for 18 h, which led to a 30% reduction of cell viability in comparison with the untreated condition (Figure 1b). The pretreatment with 2.5 µM fisetin promoted a significant protection, as revealed by the increase of cell viability compared to the *t*-BHP-treated condition (Figure 1b).

LUHMES cells differentiated into postmitotic cells recapitulate features of dopaminergic neurons, including the expression of the dopaminergic markers tyrosine hydroxylase (TH) and DAT [24]. PD is characterized by mitochondrial dysfunction leading to increased oxidative stress and by the loss dopaminergic neurons in the *substantia nigra*, which leads to a consequent decrease in the levels of dopamine [2]. MPP^+^ induces dopaminergic cell death as a consequence of its uptake via DAT and binding to the mitochondrial respiratory complex I, decreasing ATP production and increasing reactive oxygen species (ROS) [25], thereby promoting cellular events similar to those associated with PD. As so, LUHMES cells differentiated into dopaminergic neurons were used as model system of PD-associated cellular pathologies for evaluation of the protective potential of fisetin. As described above, the concentrations of fisetin tested were in the range of the physiological levels, and its nontoxicity was confirmed (Figure 1c). MPP^+^ insult strongly compromised cell viability, and preincubation with both concentrations of fisetin improved viability of MPP^+^-challenged cells (Figure 1d). 

To evaluate how fisetin-mediated protection observed in LUHMES cells was reflected in terms of dopaminergic markers, the mRNA levels of TH and DAT were monitored by qRT-PCR. TH is the limiting enzyme in the synthesis of dopamine. Decreased expression and activity of this enzyme is associated with reduced dopamine levels in dopaminergic neurons [26] and with PD pathology. As expected, MPP^+^ led to a downregulation of TH expression (Figure 1e). The decrease in TH levels due to the MPP^+^ treatment was not prevented by preincubation with fisetin. In fact, fisetin per se, reduced TH expression (Figure 1e), although it did not affect cell viability (Figure 1c). 

The dopamine transporter also plays a critical role in PD pathology, as it is responsible for reuptake of dopamine from the synaptic fence to the cell; on the other hand, DAT mediates the transport of MPP^+^ into the neurons [25]. As observed, DAT expression was not altered by fisetin preincubation in control cells but, although not significant, slightly increased after MPP^+^ treatment (Figure 1f). The importance of DAT for MPP^+^ uptake and neurotoxicity has been documented [27]. Indeed, the pretreatment with fisetin was able to significantly reduce DAT levels compared to MPP^+^ treatment, suggesting that fisetin-mediated reduction of DAT levels may help cells coping with MPP^+^ injury by decreasing its uptake. Upon MPP^+^ incubation, αsyn levels were decreased, an event that was prevented by the preincubation with fisetin (Figure 1g). 

Considering the role of αsyn in PD pathology and the fact that fisetin seems to modulate αsyn expression levels, we considered exploring what could be the effect of fisetin in αsyn-mediated processes associated with its pathological aggregation.

### 2.2. Fisetin Reduces αsyn-Mediated Toxicity in a Yeast Model of Synucleinopathy

One of the most studied features of PD pathology is the misfolding and aggregation of αsyn. Despite the neuroprotective potential of fisetin against common neurodegeneration hallmarks, its effect on αsyn aggregation and mediated toxicity was never reported before.

**Figure 2:**
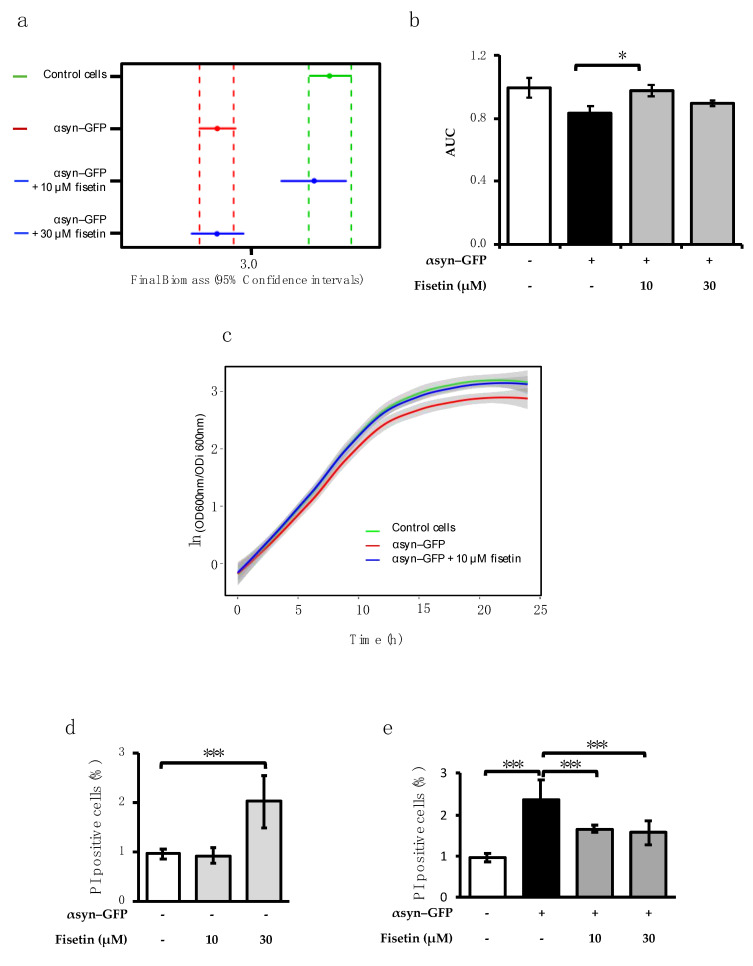
Fisetin protects against αsyn-induced growth impairment and toxicity. Control cells and cells expressing αsyn–GFP were pre-grown in raffinose medium until mid-log phase. The medium was discarded, and cells were incubated in galactose medium (αsyn ON) supplemented or not with fisetin for 24 h at 30 °C. OD_600 nm_ was monitored hourly. (**a**) Final biomass 95% confidence intervals were obtained by data modulation using nonlinear parametric regressions and were estimated from the best fit model using RStudio. The vertical dashed line represents the limits of the 95% confidence intervals. (**b**) The area under the curve (AUC) was integrated using the Origin software (OriginLab, Northampton, MA, USA). (**c**) Growth curve of control cells, αsyn–GFP-overexpressing cells, and αsyn–GFP-overexpressing cells incubated with the protective concentration of fisetin (10 µM), of which a representative image of the three biological replicates is shown. (**d**) Flow cytometry analysis of yeast cells incubated with propidium iodide to stain dead cells. Control cells incubated with 10 and 30 μM of fisetin and (**e**) cells overexpressing αsyn–GFP incubated with 10 and 30 μM of fisetin compared with control cells. The values represent the mean ± SEM of at least three biological replicates. Statistic differences are denoted by * *p* < 0.05 and *** *p* < 0.01 vs. indicated condition.

To further investigate the bioactivity of fisetin on αsyn pathology, we used a yeast model in which the overexpression of αsyn fused with green fluorescent protein (GFP) (αsyn–GFP) led to the formation of intracellular inclusions and to a reduction in cell fitness and growth [21]. We first evaluated the ability of fisetin to overcome αsyn-mediated growth impairment. For that, control cells and cells expressing αsyn–GFP were incubated with 10 or 30 µM of fisetin, and growth was monitored (Figure 2). As expected, αsyn–GFP overexpression perturbed cellular growth, impacting the final biomass of the cultures and the area under the curve (AUC) (Figure 2a,b). Remarkably, treatment of αsyn–GFP-expressing cells with 10 µM of fisetin promoted the increase in final biomass and AUC as compared to the untreated condition, unlike the treatment with 30 µM of fisetin (Figure 2a,b), which was reflected in the overall growth profile (Figure 2c). To evaluate the impact of fisetin in cellular toxicity, cell death was assessed by flow cytometry. Cells expressing the empty vector incubated with 30 µM of fisetin displayed increased toxicity (Figure 2d), an effect that was not observed for 10 µM. Both 10 and 30 µM of fisetin were found to decrease αsyn–GFP toxicity (Figure 2e). However, as 30 µM of fisetin is toxic to control cells, 10 µM was used to further investigate fisetin mechanism of action.

### 2.3. Fisetin Reduces αsyn Aggregation in a Yeast Model of Synucleinopathy

**Figure 3:**
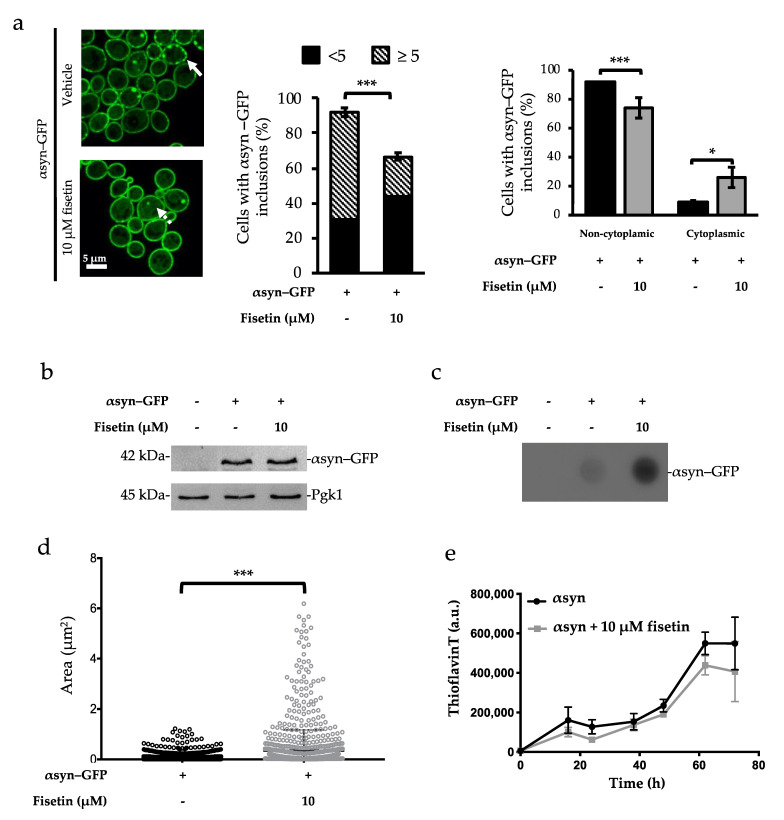
Fisetin reduces the percentage of cells with αsyn–GFP inclusions and promotes the formation of αsyn–GFP species with increased area. (**a**) Confocal microscopy imaging of αsyn–GFP-expressing cells incubated in galactose medium (αsyn ON) supplemented or not with 10 µM fisetin for 6 h at 30 °C. Scale bar 5 μm. Three biological replicates were performed, and a representative image is shown (left panel). Yeast cells containing αsyn inclusions were counted and plotted (middle panel). Yeast cells containing inclusions only in the cytoplasm (dashed arrow) or displaying inclusions in the membranes (bold arrow) were counted and plotted (right panel). (**b**) αsyn protein levels were assessed in αsyn–GFP-expressing cells incubated in galactose medium (αsyn ON), supplemented or not with 10 µM fisetin for 6 h at 30 °C. Pgk1 was used as loading control. Three biological replicates were performed, and a representative image is shown. (**c**) Filter trap assay of yeast total protein extract. Trapped αsyn–GFP species were detected using anti-αsyn antibody. (**d**) Confocal images were used to measure the area of the inclusions using Icy Spot Detector. Detection was performed for a minimum of 1000 spots on GFP channel using a 1-, 7-, and 13-pixel scale with 100, 100, and 115 sensitivity, respectively. (**e**) Fibrilization processes of αSyn was monitored. A total of 70 μM of αsyn alone or in the presence of 10 μM of fisetin was incubated at 37° under shaking. Samples were collected over time, and fibril formation was monitored by the increase in ThT fluorescence. Untreated αsyn (black filled circle), αsyn treated with 10 μM of fisetin (gray filled square). The values represent the mean ± SEM of at least three biological replicates. Statistic differences are denoted by * *p* < 0.05, *** *p* < 0.001 vs. αsyn–GFP condition.

To investigate the mechanism by which fisetin decreases αsyn–GFP growth impairment and toxicity, we assessed its effect on αsyn aggregation. Cells expressing αsyn–GFP were incubated in the presence or absence of 10 µM of fisetin, and the percentage of cells displaying αsyn–GFP inclusions was monitored (Figure 3a, left and middle panel). Cells treated with 10 µM of fisetin showed a significant reduction in the number of cells containing αsyn–GFP inclusions (Figure 3a, left and middle panel). Besides the number of cells containing αsyn–GFP inclusions, the number of aggregates per cell as well as their subcellular localization are features also associated with αsyn toxicity. It is described that the number of αsyn inclusions per cell is a parameter to have into account when searching for αsyn toxicity mechanism [28]. To assess the capacity of fisetin to modulate the number and distribution of αsyn–GFP inclusions, the number of αsyn–GFP inclusions per cell was analyzed. Interestingly, the incubation of αsyn–GFP-overexpressing cells with fisetin also promoted a decrease in the number of cells displaying less than 5 inclusions, compared with untreated αsyn–GFP overexpressing cells (Figure 3a, right panel). Concerning the subcellular localization, it is described that monomeric αsyn first localizes to the membranes. Then, as a consequence of protein accumulation, it starts to form *foci* that ultimately are released from the membrane and accumulate in the cytoplasm [21,29]. To further evaluate a possible relationship between the subcellular localization of αsyn–GFP inclusions and cytotoxicity, yeast cells displaying only cytoplasmic inclusions were counted. Interestingly, in the presence of 10 µM of fisetin, the formation of cytoplasmic inclusions was favored (Figure 3a, right panel). Thus, we suggest that fisetin decreases the percentage of cells harboring exclusively membranal αsyn inclusions, thereby avoiding cytotoxicity. 

Importantly, we assess the expression levels of αsyn–GFP (Figure 3b) and found that all the alterations in its aggregation and subcellular localization could not be attributed to alterations in protein levels.

To further assess αsyn–GFP inclusions properties, we performed filter trap assays. Upon incubation with fisetin, higher amounts of SDS insoluble αsyn–GFP species tend to be trapped in the membrane when compared with αsyn–GFP alone (Figure 3c), suggesting that fisetin promotes the formation of bigger SDS-resistant inclusions. The area of the αsyn–GFP inclusions from microscopy confocal images was also calculated using Spot Detector from Icy software (Institute Pasteur, France), and we observed that incubation with 10 µM of fisetin promoted the formation of αsyn–GFP species with a bigger area (Figure 3d). To further investigate the role of fisetin in the oligomerization process of αsyn, we allowed pure human αsyn to aggregate in vitro, alone or in the presence of 10 µM of fisetin. The presence of fisetin did not seem to impact the fibrilization process of αsyn (Figure 3e).

## 3. Discussion

Fisetin is a flavonol already described to mediate cellular protection against oxidative stress in several cellular models, including human retinal pigment epithelial cells and lung fibroblast cells [30,31]. In neuronal SH-SY5Y cells, fisetin protects from 6-hydroxydopamine (6-OHDA)-induced cell death by activating phosphatidylinositol-4,5-bisphosphate 3-kinase (PI3K)–Protein kinase B (Akt) signaling [32]. Moreover, fisetin also alleviates rotenone-induced cytotoxicity and oxidative stress in SH-SY5Y cells by downregulating apoptosis regulator BAX (Bax) and caspase-3 and by upregulating beclin 2 (Bcl-2), p38/c-Jun N-terminal kinases (JNK)–MAPK and PI3K and Akt, glycogen synthase kinase 3 beta (GSK-3β) pathways [33]. Our results reinforce this potential by showing that fisetin, at a lower concentration, and therefore more physiologically relevant in a nutritional scenario, promoted cell survival in a human neuronal cell model when submitted to oxidative insults. This activity is common to other well-described flavonols with the same backbone structure of a 3-hydroxyflavone such as quercetin, kaempferol, and myricetin already described as cytoprotective against diverse oxidative insults [34]. 

Dopaminergic neurons are the most affected neurons in PD, and it is described that MPP+ induces dopaminergic cell death. We observed a significant neuroprotective effect of fisetin in these cells upon MPP+ treatment. One of the hallmarks of PD, regarding dopaminergic neurons, is the loss of TH expression and activity. In our study, we have shown that fisetin does not prevent the decrease in TH levels imposed by MPP+ treatment in dopaminergic neurons. In fact, fisetin per se, reduced TH gene expression. However, in a recent study, it was described that fisetin is able to alleviate MPTP-induced dopaminergic neurodegeneration in the *substancia nigra*–*striatum* axis of PD mice by increasing TH protein levels and TH-positive neurons [35]. In a 6-OHDA-induced rat model of PD, it was reported that fisetin was unable to counteract TH-positive cells loss in the *substantia nigra* or to decrease the dopamine content in the *striatum* [36]. The effect of other flavonols, namely quercetin, myricetin, and kaempferol, on TH function indicates that the position and number of hydroxyl groups may play a role in the bioactivity of these compounds. Myricetin was shown to prevent 6-OHDA-mediated decrease of TH-positive cells and TH mRNA expression in the *substantia nigra* [36] and kaempferol to avoid MPTP-induced loss of TH-positive neurons in a mouse model of PD [37]. The results regarding quercetin are not conclusive, as they show a similar effect to fisetin in some studies and reversion of TH-positive cells loss in animal models of PD [38,39]. These contradictory results may indicate that alterations in TH is a secondary outcome due to neurodegeneration of dopaminergic neurons caused by yet unidentified genetic or environmental factors, and thus, TH modulation is thought to not play a direct role in PD [40]. As such, the neuroprotective potential of fisetin against MPP+ may not be directly related to the modulation of TH. Moreover, the fact that different results are obtained with different approaches and models, ranging from in vivo oral administration to in vitro direct incubation, can be a key point to the discussion of fisetin impact in TH biology. 

DAT also plays a critical role in PD, as it is responsible for reuptake of dopamine from the synaptic fence to the cell, and it is described to mediate the transport of MPP^+^ into the neurons [25]. We observed that DAT levels were not affected by the preincubation with fisetin; moreover, MPP^+^ challenge resulted in a slight but not significant increase in DAT levels, which was prevented by the preincubation with fisetin. These data may suggest a fisetin-mediated reduction of DAT levels that may contribute to prevent cells from uptaking MPP^+^. In fact, it was described that flavonoids can interfere with MPP^+^ uptake [41]. Uptake experiments in SH-SY5Y using radiolabeled MPP^+^ reveal differential effects of flavonoids. Catechin does not alter MPP^+^ uptake, whereas its metabolite 4′-methyl-catechin, epicatechin and its methylated metabolites decrease MPP^+^ uptake [41]. Otherwise, quercetin and its glucuronide increase MPP^+^ uptake [41]. Taken together, our results suggest that, by avoiding the increase in DAT levels, fisetin may contribute to the prevention of MPP^+^ internalization, thereby promoting cell survival.

Besides the central role of αsyn in PD pathology, its function remains unclear, it has been implicated in a wide range of cellular activities. It was found to regulate synaptic vesicle function [42,43] and tyrosine hydroxylase [44]. Moreover, in vitro studies of DAT have found that human αsyn overexpression can cause both a decrease or an increase in DAT activity, depending on the levels of αsyn overexpression, confirming its implication in dopamine biology [45,46,47]. To understand the role of αsyn in fisetin-mediated protection, we assessed SNCA mRNA levels in LUHMES cells. Fisetin did not result in any change in SNCA mRNA basal levels. However, preincubation with fisetin followed by MPP^+^ insult in a fisetin-mediated protective scenario prevented the decrease in SNCA mRNA levels promoted by MPP^+^. Fisetin was previously described to reduce αsyn protein levels in undifferentiated PC12 cells exposed to MPTP/MPP^+^ [18]. However, in LUHMES cells differentiated into dopaminergic neurons, a more relevant cellular model, we observed a recovery. A decrease in αsyn levels may lead to a detrimental loss of its physiological function upon MPP^+^ insult and the prevention of this event by fisetin may allow αsyn to perform its physiological role in dopamine metabolism. 

αsyn exists in equilibrium between the unfolded form in the cytoplasm and an alpha-helical-rich form when bound to membranes, but in disease conditions, it forms beta-sheet-rich amyloid fibrils that accumulate in the brain of patients [48]. The presence of αsyn toxic intracellular aggregates within the brain is the main hallmark of many neurodegenerative diseases (ND) called synucleinopathies. For this reason, efforts to decrease the presence of αsyn species are one of the main therapeutic strategies currently investigated. We demonstrated that fisetin improves cell growth and decreases toxicity overtaken by αsyn–GFP overexpression. Our results suggest that this protective effect is, in part, due to a decrease in the number of cells that harbor αsyn–GFP inclusions and in the number of αsyn–GFP inclusions per cell. Indeed, several (poly)phenols, including the flavanols quercetin, myricetin, and kaempferol flavanols, were already described as modulators of αsyn aggregation and toxicity in vitro and in cellular models [34,49]. Quercetin recovers cell growth after αsyn overexpression in yeast; however, the effect is not specific for αsyn due to its capacity to also protect cells against the toxic effects of amyloid beta (Ab) toxicity [50,51]. Moreover, (poly)phenol-enriched fractions containing flavonols were already described to reduce αsyn–GFP toxicity by promoting the formation of bigger and less toxic αsyn species. Interestingly, this (poly)phenol-enriched fraction improved cell viability by decreasing αsyn inclusions in yeast, an effect that was not mimicked in terms of toxicity by pure myricetin and quercetin glycosides. Importantly, the number of αsyn *foci* per cell was also decreased. The number of inclusions has been pointed as an indication of αsyn toxicity, being the reduction in the number of *foci* per cell an indication of decreased toxicity [52,53]. In a more nutritional relevant approach, (poly)phenols from *Arbutus unedo* L. submitted to in vitro digestion also effectively counteracted αsyn and H_2_O_2_ toxicity in yeast and human cells, improving viability by reducing αsyn aggregation [54].

Neurodegenerative diseases share similar cellular hallmarks such as the presence of protein aggregates with fibrillary amyloid-like structures in the brain. It is still an open debate if small and soluble αsyn oligomers, rather than the insoluble species, are the ones promoting cytotoxicity in these diseases, including PD. Apart from the capacity to reduce the number of cells displaying αsyn–GFP, we showed that fisetin promotes the formation of bigger intracellular inclusions but not alter the fibrilization process in vitro. This effect was already described for 3,3′,4′,5′-tetrahydroxyflavone (a synthetic analog of fisetin) on Ab 1-42, the peptide that forms amyloid-like species in AD. The fisetin analog promoted the formation of oligomers with higher molecular weight (MW) in vitro, assessed by SDS-PAGE, which are less toxic for hippocampal neurons [55]. The formation of less toxic, bigger αsyn inclusions was also observed for epigallocatechin–gallate (EGCG). The authors showed that the aggregates formed have higher MW and are spherical instead of displaying the typical fibril-like structure. They also showed that this “off-pathway” EGCG-promoted species are SDS-resistant and less toxic [56]. Furthermore, we observed that, in the presence of fisetin, the cytoplasmic, rather than membranal, localization of αsyn–GFP *foci* is favored. Indeed, it is known that the subcellular localization of αsyn aggregated species plays a role in PD pathology. Lindquist and co-workers reported that plasma membrane is a major nucleation site for αsyn aggregation, which sustains our results showing that fewer toxic conditions are associated with decreased presence of αsyn–GFP membranal inclusions. It is also described that the deposition of aggregated αsyn in perinuclear inclusions modulates its toxicity [57,58].

Fisetin protective effects against PD-related oxidative stress has been reported, but the effect of fisetin on the αsyn aggregation process were never described. In the present study, we clearly show that fisetin increases the area of the aggregates and decreases the toxicity of αsyn–GFP in a yeast model expressing human αsyn–GFP. Our data suggest that fisetin-mediated growth restoration and decreased toxicity of αsyn-expressing cells might be related to the modulation of αsyn intracellular aggregation process and localization.

Although the molecular mechanisms underlying this protective response still remains to be elucidated, our data clearly suggest that fisetin modulates αsyn inclusions dynamics by impacting their size, number, and subcellular localization. 

**Figure 4:**
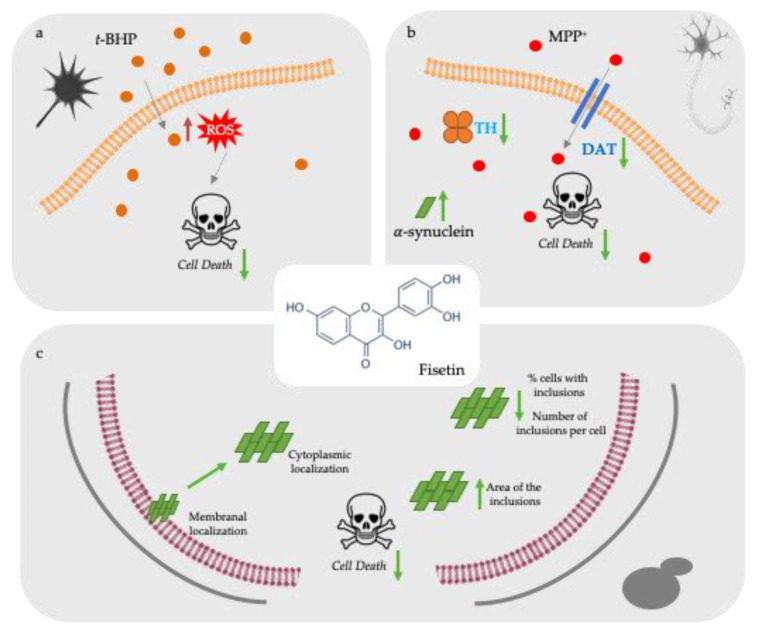
Protective activities of fisetin against PD-associated cellular pathologies. (**a**) Fisetin preincubation reduced oxidative stress-induced cell death in SH-SY-5Y cells caused by *t*-BHP lesion. (**b**) Fisetin preincubation protects cells from the dopaminergic injury caused by MPP+ with reduction of DAT and TH and an increase of SNCA mRNA expression levels. (**c**) Fisetin modulates human αsyn aggregation in a yeast model of PD. Fisetin reduces αsyn–GFP toxicity in yeast cells by decreasing the number of inclusions per cell, having these inclusions an increased area) and a predominant localization within the cytoplasm. Green arrows indicate fisetin effects in the different models.

Overall, our study unravels the protective action of fisetin against PD-associated pathological processes at nutritionally relevant concentrations in mammalian relevant cellular models (Figure 4). Additionally, and for the first time, it is suggested that fisetin affects αsyn aggregation and its subcellular localization, besides the modulation of common pathological hallmarks of NDs such as oxidative stress and attenuation of dopaminergic injury, therefore constituting a potential lead for PD therapeutics. Further studies should focus on understanding if fisetin prevents the assembly and/or promotes the removal of different αsyn structures. Also, further investigating the differential distribution of αsyn–GFP between membrane and cytoplasm, the mechanism(s) by which fisetin modulates αsyn aggregation, will be of primary importance to advance our understanding of (poly)phenol-rich foods in preventing PD. Moreover, it will be of great importance the further validation of fisetin activity using an αsyn transgenic mouse model.

Due to the widely described fisetin bioactivities, there have been increasing efforts for the development of novel delivery platforms to improve its biopharmaceutical properties. However, as a dietary phenolic with an oral bioavailability of almost 44%, our work emphasizes its potential for preventing PD pathology through nutritional interventions.

## 4. Materials and Methods

### 4.1. Reagents

Tert-butyl hydroperoxide solution (Luperox, *t*-BHP), Dulbbecco’s Modified Eagle Medium high glucose (DMEM), Eagle Minimum Essential Media (EMEM), nonessential amino acids (NEAA), sodium pyruvate, L-glutamine, poly-L-ornithine, human plasma fibronectin, tetracycline, cAMP, 1-Methyl-4-phenylpyridinium dihydrochloride (MPP^+^), D(+)-Glucose, D-(+)-Raffinose, D(+)-Galactose, glass beads, Ethylenediaminetetraacetic acid (EDTA), trypsin from bovine pancreas and fisetin were purchased from Sigma Aldrich Co., Ltd. (St. Louis, MO, USA). Heat-inactivated fetal bovine serum (FBS) and trypsin/EDTA were purchased from Gibco (Carlsbad, CA, USA). CellTiter Blue^®^ Cell Viability Assay was purchased from Promega, (Madison, WI, USA). Advanced DMEM/F12 media and N2-supplement were purchased from Invitrogen Corporation (Carlsbad, CA, USA). Fibroblast growth factor (FGF) and glial-derived neurotrophic factor (GDNF) were purchased from R&D System Inc. (Minneapolis, MN, USA). Complete Synthetic Medium was purchased from MP Biomedicals (Santa Ana, CA, USA). Yeast Nitrogen Base without amino acids and monoclonal mouse anti-αsyn antibody (clone 42) were purchased from BD Biosciences (San Jose, CA, USA). Mouse monoclonal anti-phosphoglycerate kinase (PGK) was purchased from Life Technologies Corporation. Light-Cycler 480 SYBR Green I Master Kit, Roche High Pure RNA Isolation Kit, Roche Transcriptor First Strand cDNA Synthesis kit were purchased from Roche (F. Hoffmann-La Roche, Basel, Switzerland). Sodium chloride was purchase from Panreac (Barcelona, Spain). Sodium hydrogen carbonate was purchased from VWR Chemicals (Radnor, PA, USA). Potassium chloride was purchased from Merck (Kenilworth, NJ, USA).

### 4.2. Cell Culture Conditions and Treatments

Human neuroblastoma cell line (SH-SY5Y–ATCC^®^ CRL-2266™) was obtained from American Type Cell Culture (ATCC) and cultured in T75 flask Nunc™ (Thermo Scientific, Walthman, MA, USA), with DMEM supplemented with 10% (*v/v*) heat-inactivated FBS, and 1 mM sodium pyruvate. Cells were maintained at 37 °C in 5% (*v/v*) CO_2_ and subcultured at confluence of 80% (every 3–4 days) using 0.05% trypsin/EDTA. To evaluate neuroprotective effects of fisetin, 1.25 × 10^5^ cells mL^−1^ were preincubated for 6 h with 2.5 µM of fisetin. The medium was discarded, and cells were washed with phosphate buffer saline (PBS) prior to addition of fresh medium containing 35 µM of *t-*BHP solution for 18 h. CellTiter Blue^®^ Cell Viability Assay was added according to the manufacturer’s instructions, and fluorescence was read at 560 nm (SynergyHT micro plate reader, BIOTEK, Winooski, VT, USA).

Lund human mesencephalic cells (LUHMES) were maintained at 37 °C in 5% (*v/v*) CO_2_ and cultured in proliferation media: advanced DMEM/F12 media supplemented with L-glutamine (200 mM), N2-supplement (100×), and FGF (160 µg mL^−1^). T Flask™ and plates (Nunc, Thermofisher Scientific, Waltham, MA, USA) were coated overnight at 37 °C with poly-L-ornithine (50 mg mL^−1^) and human plasma fibronectin (l mg mL^−1^). LUHMES cells were differentiated by medium alteration: proliferation medium was replaced by differentiation medium (DMEM/F12 supplemented with 200 mM of L-glutamine, N2 supplement, l mg mL^−1^ tetracycline, 500 mM cAMP, and 20 µg mL^−1^ GDNF). After 48 h, cells were trypsinized and seeded into plates. Cells were maintained with differentiation medium during 72 h before experiment start. To evaluate neuroprotective effects of fisetin in a PD model (0.05 × 10^6^ cell/well), LUHMES cells were preincubated during 24 h with 1.25 and 2.5 µM of fisetin. The medium was discarded, and differentiation medium, without cAMP and GDNF [59,60], containing 5 µM of MPP^+^ was added for 24 h. Cell viability was assessed by CellTiter-Blue^®^ Cell Viability Assay according to the manufacturer’s instructions. Fluorescence was read at 560 nm (SynergyHT micro plate reader).

### 4.3. Quantitative Real-Time PCR

Quantitative real-time PCR (RT-qPCR) was performed as described [4]. LUHMES cells, at a cellular density of 0.55 × 10^6^ cell/well, were grown and differentiated into 12-well plates. Cells were harvested, snap-frozen, and stored at −80 °C until use. RNA was extracted using Roche High Pure RNA Isolation Kit, following the manufacturer’s instructions, and quantified using the Nanodrop ND-2000C (ThermoScientific, Waltham, MA USA). cDNA Synthesis was performed using Roche Transcriptor First Strand cDNA Synthesis kit according to the manufacturer’s instructions. cDNA was diluted 1:50, and quantification of TH and DAT mRNA levels was performed in the Light Cycler 480 Multiwell Plate 96 (Roche) using the Light-Cycler 480 SYBR Green I Master Kit (Roche) and the oligonucleotide primers listed in Table 1 at a final concentration of 5 µM. Reactions were performed in duplicate in a final volume of 20 µL. Cycle’s threshold (Ct’s) and melting curves were determined using Light Cycler 480 software, version 1.5 (Roche), and results were processed using relative quantification method for relative gene expression analysis [4]. Gene expression data were normalized using HPRT1, GAPDH, and B2M as internal controls.

### 4.4. Yeast Strains and Growth Conditions

The strains W303-1A *trp1-1::pRS304 TRP1 ura3-1::pRS306 URA3* and W303-1A_ αsyn *trp1-1::pRS304 GAL1pr-SNCA(WT)-GFP TRP1 ura3-1::pRS306 pRS306GAL1pr-SNCA(WT)-GFP::URA3*, expressing the empty constructions and a double genomic insertion of GAL1prSNCA(WT)-GFP, respectively, were previously described [21]. 

For all experiments, cells were grown in liquid Synthetic Complete medium (SC)–raffinose [0.67% yeast nitrogen base without amino acids (YNB), 0.79 g L^−1^ complete supplement mixture (CSM), 1% raffinose] for 24 h at 30 °C and under orbital agitation (200 rpm). The optical density at 600 nm (OD_600 nm_) was measured, and cell cultures were diluted to OD_600 nm_ 0.2 in SC–raffinose and incubated for further 24 h under the same conditions. 

### 4.5. Growth Curves

Fisetin bioactivity in the PD yeast model was monitored by growth curves, as previously described [28]. After incubation in SC–raffinose, OD_600 nm_ was monitored, and a fresh inoculum to a final OD_600 nm_ 0.04 ± 0.004 was performed in SC–galactose [0.67% YNB, 0.79 g L^−1^ CSM, 2% galactose]. The fresh cultures were transferred to 96-well plates and incubated for 24 h at 30 °C and under orbital agitation in the PowerWave XS microplate reader (Biotek) in media supplemented or not with nontoxic concentrations fisetin (10 and 30 µM). Data was modeled using nonlinear parametric regressions, and growth parameters (final biomass, maximum growth rate, lag time, and doubling time), all with 95% confidence intervals, were estimated from the best fit model using Rstudio (Rstudio Version 0.99.902, GNU lesser general public license, Boston, MA, USA), as previously described [61]. The area under the curve (AUC) was integrated using the Origin software (OriginLab, Northampton, MA, USA).

### 4.6. Fluorescence Microscopy

After 24 h incubation in SC–raffinose, OD_600 nm_ was adjusted, and cells were incubated overnight. Cells corresponding to an OD_600 nm_ 0.2 ± 0.02 were incubated in SC–galactose medium supplemented or not with fisetin (10 μM and 30 μM). After 6 h incubation, cells were collected by centrifugation, resuspended in PBS, and the microscopy slides were prepared. The percentage of cells containing αsyn–GFP inclusions was determined by fluorescence microscopy using a Zeiss LSM 710 confocal microscope system with a Zeiss AXIO Observer Z1 inverted microscope stand with transmitted (HAL), UV (HBO), and laser illumination sources (Zeiss, Oberkochen, Germany). At least 400 cells per condition were manually counted using ImageJ software (NIH, Bethesda, MD, USA). The area of the inclusions was calculated using Spot Detector from Icy software (Institute Pasteur, Paris, France). Detection was performed for a minimum of 1000 spots on GFP channel using a 1-, 7-, and 13-pixel scale with 100, 100, and 115 sensitivity, respectively. 

### 4.7. Flow Cytometry Analysis

After 24 h incubation in SC–raffinose, OD_600 nm_ was adjusted, and cells were incubated overnight. Cells corresponding to an OD_600 nm_ 0.2 ± 0.02 were incubated in SC–galactose medium supplemented or not with fisetin (10 µM and 30 µM). After 6 h incubation, cells were collected. Flow cytometry analysis were performed in a FACS BD Canto II equipped with the laser. To determine cell viability with PI, cells were incubated with 5 μg mL^−1^ of PI for 30 min, protected from light. Data analysis was performed using FlowJo software; 30,000 events were collected for each experiment. Results were expressed as a percentage of PI-positive cells.

### 4.8. Protein Analysis

After 24 h incubation in SC–raffinose, OD_600 nm_ was adjusted, and cells were incubated overnight. Cells corresponding to an OD_600 nm_ 0.2 ± 0.02 were incubated in SC–galactose medium supplemented or not with 10 μM fisetin. After 6 h incubation, cells were collected by centrifugation and lysed in MURB buffer (50 mM sodium phosphate, 25 mM MES pH 7.0, 1% sodium dodecyl sulfate (SDS), 3 M urea, 0.5% 2-mercaptoethanol, 1 mM sodium azide, supplemented with protease and phosphatase inhibitors (Roche, Mannheim, Germany)) with glass beads. Protein samples were heated at 70 °C for 10 min and centrifuged at 10,000× *g* for 1 min before gel loading. Equal volumes of protein extract, normalized to the OD_600 nm_ of cell cultures, were loaded in a 15% SDS–PAGE. Proteins were transferred to 0.22 µm nitrocellulose membranes using a Trans-Blot Turbo transfer system (BioRad, Hercules, CA, USA), following manufacturer’s specifications. Immunoblotting was performed following standard procedures using anti-αsyn antibody. Pgk1 was used as loading control. Images were acquired using Chemidoc™ XRS and ImageLab^®^ software.

### 4.9. Filter Trap Analysis

After 24 h incubation in SC–raffinose, OD_600 nm_ was adjusted, and cells were incubated overnight. Cells corresponding to an OD_600 nm_ 0.2 ± 0.02 were incubated in SC–galactose medium supplemented or not with 10 μM fisetin. After 6 h incubation, cells were collected by centrifugation, and total protein was extracted as previously described [53].

For filter trap analysis, 100 μg of total protein were diluted in TBS 1% (*v/v*) SDS and loaded onto a pre-equilibrated nitrocellulose membrane (0.22 μm) (GE Healthcare, Chicago, IL, USA) in a slot blot apparatus. The samples were allowed to pass through the membrane by vacuum, and the slots were washed twice with TBS 1% (*v/v*) SDS. Immunoblotting was performed following standard procedures using anti-αsyn antibody. Images were acquired using Chemidoc™ XRS and ImageLab^®^ software.

### 4.10. Thioflavin T Assay

Pure human αsyn was purchased from rPeptide (Watkinsville, GA, USA). Pure αsyn was reconstituted using 50 mM Tris–HCl buffer (pH 7.4). For the aggregation assay, the protein solution (70 μM) was mixed with a final concentration of 10 μM of fisetin in 50 mM Tris–HCl buffer (pH 7.4). Protein samples were stirred at 900 rpm at 37 °C in a thermomixer (Eppendorf, Hamburg, Germany). Then, 1.4 μM of αsyn was added to 1 μM of ThT solution in 50 mM Tris–HCl buffer, and ThT fluorescence was recorded at the indicated time points, as described [28].

### 4.11. Statistical Analysis

Statistical analysis was carried out using Graphpad Prism 6 software. Data are mean ± SEM of at least three independent biological replicates. One-way ANOVA with the Tukey HSD (honest significant difference) multiple comparison test (α = 0.05) was performed to access differences between the conditions. For qRT-PCR, *t*-Test was performed to access differences between the conditions.

## Figures and Tables

**Table 1:** List of oligonucleotide primers used for RT-qPCR analysis.

Gene	Nomenclature	Sequence
***TH***	Tyrosine hydroxylase	Fwd ^1^: AGCCCTACCAAGACCAGACG Rev ^1^: GCGTGTACGGGTCGAACTT
***DAT***	Dopamine transporter	Fwd: ACCTTCCTCCTGTCCCTGTT Rev: CACCATAGAACCAGGCCACT
***SNCA***	Alpha-synuclein	Fwd:AGTGACAAATGTTGGAGGAG Rev: GCTTCAGGTTCGTAGTCTTG
***HPTR1***	Hypoxanthine phosphoribosyltransferase 1	Fwd: CCTGGCGTCGTGATTAGTGA Rev: CGAGCAAGACGTTCAGTCCT
***GAPDH***	Glyceraldehyde 3-phosphate dehydrogenase	Fwd: AGAAGGCTGGGGCTCATTTG Rev: AGGGGCCATCCACAGTCTTC
***B2M***	β2 microglobulin	Fwd: GGCTATCCAGCGTACTCCAA Rev: ACCAGTCCTTGCTGAAAGACAA

^1^ Fwd—forward; Rev—reverse.

## Data Availability

Not applicable.

## Sample Availability

Samples of the compounds are not available from the authors.
